# Moderate physical activity may not decrease the risk of cardiovascular disease in persistently overweight and obesity adults

**DOI:** 10.1186/s12967-021-03212-7

**Published:** 2022-01-28

**Authors:** Qiuyue Tian, Biyan Wang, Shuohua Chen, Shouling Wu, Youxin Wang

**Affiliations:** 1 Beijing Key Laboratory of Clinical Epidemiology, School of Public Health, Capital Medical University, 10 YouanmenXitoutiao, Beijing, 100069 China; 2 Department of Cardiology, Kailuan General Hospital, North China University of Science and Technology, 57 Xinhua East Road, Tangshan, 063000 China

**Keywords:** Cardiovascular disease, Body mass index, Physical activity, Long-term trajectories, All-cause mortality

## Abstract

**Background:**

Body mass index (BMI) and physical activity (PA) has been documented to be associated with cardiovascular disease (CVD). However, the evidences regarding joint phenotypes of BMI and PA trajectories with risk for CVD and all-cause mortality are still limited.

**Methods:**

Participants from the Kailuan Study, followed up during 2006–2019 were included, with primary outcomes of CVDs (myocardial infarction or stroke) and all-cause mortality. BMI and PA were repeatedly measured at least three times, and thus joint phenotypes trajectory groups were identified by group-based trajectory modeling. Cox proportional hazards models were used to examine the associations between trajectory groups and CVDs and all-cause mortality.

**Results:**

Totally 88,141 (6 trajectories) and 89,736 participants (5 trajectories) were included in the final analyses relating trajectories to CVDs and all-cause mortality, respectively. Compared with persistent normal-weight with moderate PA group, participants were associated with increased risk of CVD in persistent overweight with moderate PA trajectory group (adjusted hazard ratio [aHR]: 1.31, 95% confidence interval [CI]: 1.22–1.41) and persistent obesity with moderate PA trajectory group (aHR: 1.55, 95% CI: 1.41–1.69). While the rising to overweight with moderate PA in normal-weight status with active PA (aHR: 0.72, 95% CI: 0.65–0.79), persistent overweight with moderate PA (aHR: 0.92, 95% CI: 0.87–0.97) and decline to normal-weight in overweight status with moderate PA (aHR: 0.73, 95% CI: 0.67–0.80) trajectories group were significantly associated with decreased all-cause mortality risk. The associations remained robust among stratifying by age and sex individuals and sensitive analysis.

**Conclusions:**

The long-term trajectories analysis showed that moderate PA may not decrease the risk of CVD in persistently overweight and obesity adults.

**Supplementary Information:**

The online version contains supplementary material available at 10.1186/s12967-021-03212-7.

## Background

In the worldwide, cardiovascular diseases (CVDs) is the leading cause of mortality with a major contributor to disability, according to the Global Burden of Disease Study 2019 [1, 2]. CVDs, principally ischemic heart disease and stroke, account for 40% of the cause of deaths in the Chinese population [3]. The dramatic increase in obesity and overweight prevalence was caused by changes in dietary and physical activity patterns, especially during the past two decades of rapid economic growth in China [4, 5].

The relationship of obesity and CVDs has been widely discussed over recent years. Most epidemiological evidences supporting a link between obesity and CVDs are based on one time point assessment [6, 7]. In addition, obesity was also significantly associated with higher all-cause mortality [8, 9]. However, despite these adverse effects on increasing the risk of CVDs, numerous studies demonstrated the obesity paradox, where CVDs patients with overweight and obesity seem to have a survival advantage [10–12]. Previous research has highlighted the need to take into account the time-varying nature of obesity and changes in weight status over the lifespan to understand the true relationship of obesity and disease risk [13]. Although body mass index (BMI) is widely acceptable to evaluate weight status and related disease risk, a simple classification of overweight and obesity by BMI at one point time may not be sufficient to explain long-term weight. Moreover, considering longitudinal changes in obesity and their associations with CVDs and mortality may help direct us toward a more meaningful clinical question, because it provides insight as to whether intervening on the BMI pathway is in fact able to provide a survival benefit.

Physical activity (PA) is closely related to obesity, and any comprehensive study focused on obesity should take PA into account. Several studies have confirmed that PA is related to lower risk of CVDs and longer life expectancy [14, 15]. In addition, studies also showed that participants with overweight or obesity but active PA were not associated with a higher CVDs risk [16, 17]. However, a recent study involving 527,662 participants showed that obesity or overweight was associated an increased risk of CVDs regardless of PA levels, which similar with several cohort studies [18–20]. Thus, it is necessary to investigate the association between long-term trajectory of joint PA-BMI phenotypes and outcomes.

In the study, we used the group-based trajectory model (GBTM) to identify distinct trajectories of joint phenotypes of obesity and PA and to examine the associations between these long-term variation trajectories and risks of CVDs (including myocardial infarction (MI) and stroke) and all-cause mortality.

## Methods

### Study design and participants

We performed a population-based retrospective longitudinal cohort study based on data from the Kailuan study, which is an ongoing prospective study in Tangshan, China. Briefly, from June 2006 to October 2007, 101,510 individuals (including 81,110 men and 20,400 women, aged 18–98 years) in the Kailuan community were enrolled to participate in the study, and followed up biennially [21–24]. In this study, we identified the joint phenotypes trajectories using joint phenotypes status in 2006, 2008, 2010, 2012, 2014, 2016, and used these patterns to predict incident CVDs and all-cause mortality. Therefore, we excluded participants who did not have data for BMI (or height and weight) and PA at baseline, have baseline BMI less than 18.5 kg/m^2^, and have history of CVDs at baseline. In addition, we further excluded participants with less than 3 times repeatedly measurement of joint BMI-PA phenotypes status due to loss to follow-up including occurring outcomes or other reasons during 2006 to 2016, respectively. This study was approved by the ethics committees of Kailuan General Hospital. Written informed consent form was obtained from all participants.

### Assessment of the joint phenotypes at baseline

BMI was calculated as weight in kilograms divided by the square of height in meters. According to the Chinese-specific criteria, normal weight (NW) were defined as 18.5 ≤ BMI < 24.0 kg/m^2^, overweight (OW) were defined as 24.0 ≤ BMI < 28.0 kg/m^2^, and obesity were defined as BMI ≥ 28 kg/m^2^ [25]. Participants were divided into three groups: inactive (< 1 time/week), moderate (1–2 times/week), and active (≥ 3 times/week and ≥ 30 min/time) according to the self-reported the frequency of PA from questionnaire [26]. Then, at baseline participants were categorized into nine joint phenotypes groups: NW and active PA (NWAPA), NW and moderate PA (NWMPA), NW and inactive PA (NWIPA), OW and active PA (OWAPA), OW and moderate PA (OWMPA), OW and inactive PA (OWIPA), obesity and active PA (OAPA), obesity and moderate PA (OMPA), obesity and inactive PA (OIPA).

### Assessment of the joint phenotypes trajectories

In this study, we evaluated the joint BMI-PA phenotypes status among participants from 2006 to 2016. On the basis of no less than three values of joint BMI-PA phenotypes status, we used the GBTM to identify subgroups, which shared similar underlying trajectories [27–29]. Model fit was assessed using the Akakike information criteria (AIC), Bayesian information criterion (BIC), and Average posterior probability (AvePP) [28]. We initiated a model with one trajectory and then compared the model fit index to that with two, three, four, five, six, seven, eight, and nine, respectively. Then, cubic, quadratic, and linear terms were considered and evaluated based on their significance level, starting with the highest polynomial. In our final model, 6 trajectories with cubic order terms and 5 trajectories with cubic order terms as the best fit were included in the further analyses relating trajectories to CVDs and all-cause mortality respectively.

### Follow-up and outcomes

All participants were followed up every two years until death or December 31, 2019. The primary outcomes were CVDs (include stroke and MI) and all-cause mortality. The diagnoses information on CVDs was obtained from medical records from medical insurance or hospitals [21, 23]. Death diagnosis information comes from family report, death certificates from provincial vital statistics offices, and medical records from medical insurance or hospitals [21, 23].

### Covariates

Socio-demographic data, lifestyle factors and medical history were collected via standardized questionnaires and clinical examinations at baseline and follow-up [4, 21, 23]. We selected age, sex, types of work, seat time, walking instead of the elevators, educational level, smoking status, drinking status, family per-member monthly income, salt intake, drinking tea status, C-reactive protein (CRP), and history of diseases (hypertension, diabetes, and hyperlipidemia) as covariates. Each covariate was collected during biennially follow-up.

### Statistical analyses

Continuous variables were described as median with interquartile range (IQR), and categorical variables were showed as number (percentage). The comparisons of continuous or categorical variables were conducted using the Kruskal–Wallis test or chi-square tests, respectively and the trend association was estimated using linear regression or Chi-square trend tests.

Person-years were calculated from the date of baseline examination to the date of CVDs or death, or the end of follow-up (December 31, 2019), whichever came first. The adjusted cumulative incidence of CVDs or all-cause mortality was estimated using Kaplan–Meier method and compared by log-rank. The proportional hazards assumption was tested by the Schoenfeld residuals, and no violation was found. Associations between trajectory groups and the risk of CVDs and all-cause mortality were estimated by cox proportional hazards regression models with 95% confidence intervals (CIs). We fitted 3 models. Model 1 was a crude model without adjusted covariates. Model 2 was adjusted for age, sex, types of work, seat time, and walking instead of the elevators. Model 3 was further adjusted for educational level, smoking status, drinking status, family per-member monthly income, salt intake, drinking tea status, CRP, and history of diseases (hypertension, diabetes, and hyperlipidemia).

To test the robustness of the main results, several sensitivity analyses were performed. Considering non-CVDs death as a competing risk event rather than a censoring event, Fine-Gray competing risk model was applied to address this issue. Considering the time dependence of variables, time-dependent cox proportional hazards models were constructed to explore the associations between trajectory groups and the risk of CVDs and all-cause mortality, while simultaneously adjusting for time-varying confounders and other covariates. Likelihood ratio test was conducted to examine statistical interactions among trajectory groups, sex, and age (< 65 years and ≥ 65 years) in association with CVDs and all-cause mortality by comparing − 2 log likelihood chi-square between nested models, with or without the multiplication interaction terms.

All statistical analyses were conducted using SAS, version 9.4 (SAS Institute Inc). Two-sided *P* < 0.05 was considered statistically significant.

## Results

**Fig. 1:**
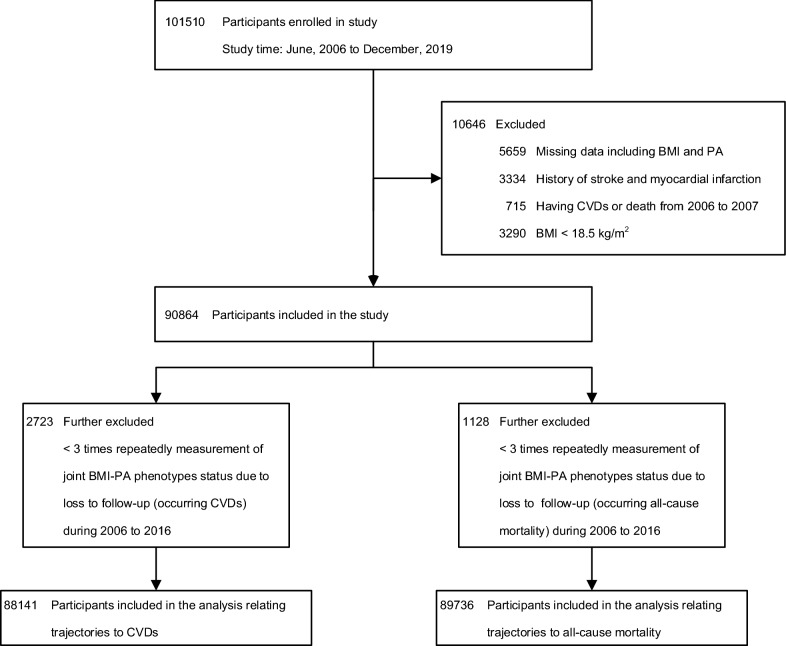
The flow diagram of study participants. *BMI* body mass index, *CVDs* cardiovascular diseases, *PA* physical activity

Of 101,510 participants were enrolled, 10,646 participants were excluded due to missing information on BMI or PA (*n* = 5659), history of stroke or MI (*n* = 3334), CVDs at baseline (*n* = 715), and BMI of less than 18.5 kg/m^2^ (*n* = 3290). In addition, we excluded participants with less than 3 times repeatedly measurement of joint BMI-PA phenotypes status due to loss to follow-up including occurring CVDs (*n* = 2723) or all-cause mortality (*n* = 1128) during 2006 to 2016, respectively (Fig. 1). Finally, the trajectories of joint BMI-PA phenotypes and their association with future CVDs and all-cause mortality risk were examined among the remaining 88,141 (accounting for 97% of samples) and 89,736 participants (accounting for 98.76% of samples), respectively.

**Fig. 2:**
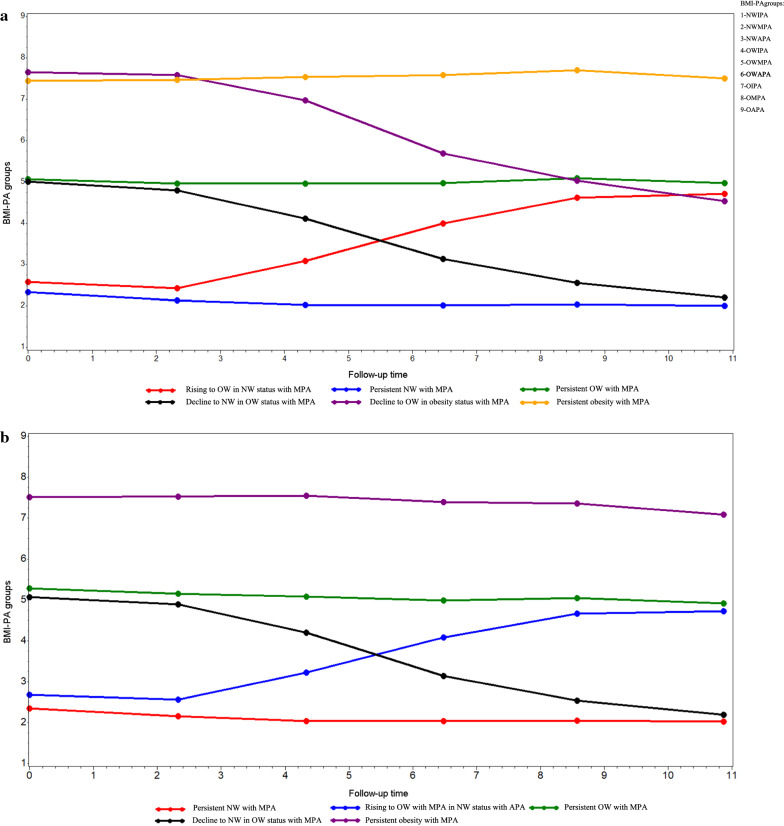
Trajectory of joint BMI-PA phenotypes during 2006–2016. The joint BMI-PA groups over time for subgroups of participants clustered according to group-based trajectory model (GBTM) estimation are shown. GBTM was produced using Proc Traj procedure in SAS 9.4 software. BMI-PA groups include NWIPA, NWMPA, NWAPA, OWIPA OWMPA, OWAPA, OIPA, OMPA, and OAPA. **a** Trajectory of joint BMI-PA phenotypes during 2006–2016 for CVDs outcome. **b** Trajectory of joint BMI-PA phenotypes during 2006–2016 for all-cause mortality outcome. *APA* active physical activity, *BMI* body mass index, *CVDs* cardiovascular diseases, *MPA* moderate physical activity, *NW* normal-weight, *NWIPA* normal weight and inactive physical activity, *NWMPA* normal weight and moderate physical activity, *NWAPA* normal weight and active physical activity, *OW* overweight, *OWIPA* overweight and inactive physical activity, *OWMPA* overweight and moderate physical activity, *OWAPA* overweight and active physical activity, *OIPA* obesity and inactive physical activity, *OMPA* obesity and moderate physical activity, *OAPA* obesity and active physical activity

We categorized the study population into six observed discrete trajectories to predict incident CVDs (Fig. 2a): 34.13% of participants who remained within the OWMPA range during 11 follow-up years (referred to as “persistent OW with moderate active physical activity (MPA)” group); 31.35% of the participants who remained within the NWMPA range during 11 follow-up years (referred to as “persistent NW with MPA” group); 13.46% of the participants who remained within the OMPA range during 11 follow-up years (referred to as “persistent obesity with MPA” group); 8.29% of participants who started with NWMPA status and experienced an increase from NW to OW with MPA (referred to as “rising to OW in NW status with MPA” group); 7.87% of the participants who started with OWMPA status and experienced an decrease from OW to NW with MPA (referred to as “decline to NW in OW status with MPA” group); and 4.90% of the participants who started with OMPA status and experienced an decrease from obesity to OW with MPA (referred to as “decline to OW in obesity status with MPA” group). Similarly, we categorized the study population into five observed discrete trajectories to predict all-cause mortality (Fig. 2b): 36.00% of participants who remained within the OWMPA range during 11 follow-up years (referred to as “persistent OW with MPA” group); 31.65% of participants who started with NWAPA status and experienced an increase from NW to OW and a decrease from active physical activity (APA) to MPA (referred to as “rising to OW with MPA in NW status with APA” group); 15.96% of the participants who remained within the OMPA range during 11 follow-up years (referred to as “persistent obesity with MPA” group); 8.75% of the participants who remained within the NWMPA range during 11 follow-up years (referred to as “persistent NW with MPA” group); 7.64% of the participants who started with OWMPA status and experienced an decrease from OW to NW with MPA (referred to as “decline to NW in OW status with MPA” group). The baseline characteristics of participants according trajectory groups are presented in Additional file 1: Table S1, S2. Trajectory groups with obesity were more likely to be had higher proportion of older individuals, history of disease (hypertension, diabetes, and hyperlipidemia), and tea drinker, longer seat time, higher level of salt intake, lower high-density lipoprotein cholesterol (HDL-C), and higher blood pressure, waist circumference (WC), hip circumference (HC), triglycerides (TG), total cholesterol (TC), fasting blood glucose (FBG), CRP, and low-density lipoprotein cholesterol (LDL-C), compared with other trajectory groups.

**Fig. 3:**
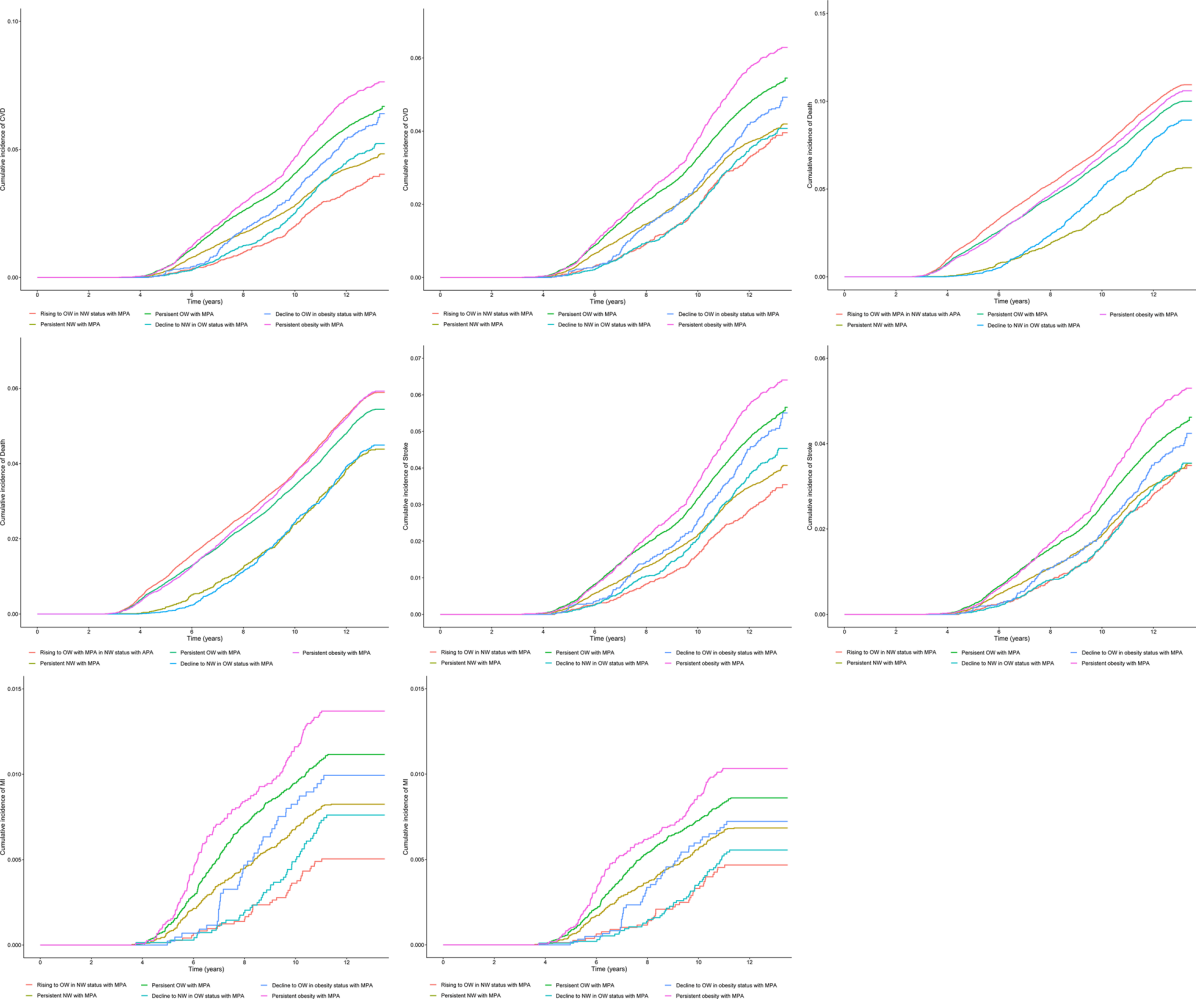
The cumulative incidence of CVDs and all-cause mortality according to the trajectory groups. The unadjusted (**a**) and adjusted (**b**) cumulative incidence of CVDs according to the trajectory groups; The unadjusted (**c**) and adjusted (**d**) cumulative incidence of all-cause mortality according to the trajectory groups; The unadjusted (**e**) and adjusted (**f**) cumulative incidence of stroke according to the trajectory groups; The unadjusted (**g**) and adjusted (**h**) cumulative incidence of MI according to the trajectory groups. CVDs included MI and stroke. Model adjusted potential confounding factors, including age, sex, type of work, seat time, walking instead of the elevators, educational level, smoking status, drinking status, family per-member monthly income, salt intake, drinking tea status, C-reaction protein, and history of diseases (hypertension, diabetes, and hyperlipidemia). *APA* active physical activity, *CVDs* cardiovascular diseases, *MI* myocardial infarction, *MPA* moderate physical activity, *NW* normal-weight, *OW* overweight

**Fig. 4:**
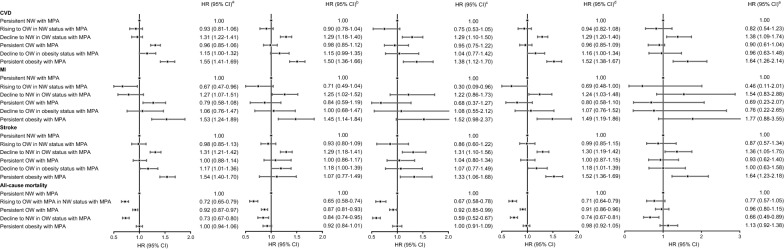
The associations of the trajectory groups with CVDs and all-cause mortality. The associations of the trajectory groups with CVDs and all-cause mortality in overall (**a**); in age < 65 years (**b**); in age ≥ 65 years (**c**); in men (**d**); in women (**e**). CVDs included MI and stroke. Multivariate cox regression analysis was used after adjusted for educational level, smoking status, drinking status, family per-member monthly income, salt intake, drinking tea status, C-reaction protein, and history of diseases (hypertension, diabetes, and hyperlipidemia). *APA* active physical activity, *CI* confidence interval, *CVDs* cardiovascular diseases, *HR* hazard ratio, *MI* myocardial infarction, *MPA* moderate physical activity, *NW* normal-weight, *OW* overweight

The unadjusted and adjusted cumulative incidence of CVDs and all-cause mortality according to the trajectory groups (Fig. 3). After adjusting covariates, persistent OW with MPA (HR: 1.31, 95% CI: 1.22–1.41), decline to OW in obesity status with MPA (HR: 1.15, 95% CI: 1.00–1.32), and persistent obesity with MPA (HR: 1.55, 95% CI: 1.41–1.69) were associated with an increased risk of CVDs compared with persistent NW with MPA group (Fig. 4; Table 1). Rising to OW with MPA in NW status with APA (HR: 0.72, 95% CI: 0.65–0.79), persistent OW with MPA (HR: 0.92, 95% CI: 0.87–0.97), and decline to NW in OW status with MPA (HR: 0.73, 95% CI: 0.67–0.80) were associated with a decrease risk of all-cause mortality (Fig. 4; Table 1).

**Table 1:** Associations of the trajectory groups with CVDs and all-cause mortality^a^

	Number^b^	Rate (‰)^c^	Model 1^e^	*P*	Model 2^f^	*P*	Model 3^g^	*P*
			HR (95% CI)		HR (95% CI)		HR (95% CI)	
CVDs^d^								
Persistent NW with MPA (reference)	1208	3.81	–	–	–	–	–	–
Rising to OW in NW status with MPA	275	3.20	0.83 (0.73–0.94)	0.0042	0.94 (0.82–1.07)	0.3424	0.93 (0.81–1.06)	0.2701
Persistent OW with MPA	1836	5.33	1.40 (1.30–1.50)	< 0.0001	1.37 (1.27–1.47)	< 0.0001	1.31 (1.22–1.41)	< 0.0001
Decline to NW in OW status with MPA	338	4.17	1.08 (0.96–1.22)	0.1977	1.00 (0.88–1.13)	0.9688	0.96 (0.85–1.06)	0.5168
Decline to OW in obesity status with MPA	252	5.00	1.30 (1.13–1.49)	0.0002	1.24 (1.08–1.42)	0.0022	1.15 (1.00–1.32)	0.0494
Persistent obesity with MPA	843	6.23	1.64 (1.50–1.79)	< 0.0001	1.69 (1.55–1.85)	< 0.0001	1.55 (1.41–1.69)	< 0.0001
MI								
Persistent NW with MPA (reference)	217	3.81	–	–	–	–	–	–
Rising to OW in NW status with MPA	36	3.20	0.61 (0.43–0.86)	0.0055	0.68 (0.48–0.98)	0.0366	0.67 (0.47–0.96)	0.0299
Persistent OW with MPA	323	5.33	1.36 (1.15–1.62)	0.0005	1.33 (1.12–1.58)	0.0012	1.27 (1.07–1.501)	0.0076
Decline to NW in OW status with MPA	51	4.17	0.91 (0.67–1.24)	0.5447	0.84 (0.62–1.14)	0.2594	0.79 (0.58–1.08)	0.1412
Decline to OW in obesity status with MPA	42	5.00	1.20 (0.86–1.67)	0.2764	1.15 (0.82–1.60)	0.4151	1.06 (0.76–1.47)	0.7465
Persistent obesity with MPA	156	6.23	1.67 (1.36–2.05)	< 0.0001	1.71 (1.39–2.11)	< 0.0001	1.53 (1.24–1.89)	< 0.0001
Stroke								
Persistent NW with MPA (reference)	1008	3.81	–	–	–	–	–	–
Rising to OW in NW status with MPA	240	3.20	0.86 (0.75–1.00)	0.0420	0.99 (0.86–1.13)	0.8327	0.98 (0.85–1.13)	0.7419
Persistent OW with MPA	1539	5.33	1.40 (1.29–1.52)	< 0.0001	1.37 (1.27–1.49)	< 0.0001	1.31 (1.21–1.42)	< 0.0001
Decline to NW in OW status with MPA	291	4.17	1.12 (0.98–1.27)	0.0920	1.03 (0.91–1.18)	0.6309	1.00 (0.88–1.14)	0.9831
Decline to OW in obesity status with MPA	214	5.00	1.32 (1.14–1.53)	0.0002	1.26 (1.09–1.46)	0.0022	1.17 (1.01–1.36)	0.0402
Persistent obesity with MPA	701	6.23	1.63 (1.48–1.80)	< 0.0001	1.68 (1.53–1.85)	< 0.0001	1.54 (1.40–1.70)	< 0.0001
All-cause mortality								
Persistent NW with MPA (reference)	3072	9.35	–	–	–	–	–	–
Rising to OW with MPA in NW status with APA	482	5.19	0.55 (0.50–0.60)	< 0.0001	0.73 (0.66–0.80)	< 0.0001	0.72 (0.65–0.79)	< 0.0001
Persistent OW with MPA	3198	8.52	0.91 (0.86–0.95)	0.0001	0.95 (0.90–1.00)	0.0335	0.92 (0.87–0.97)	0.0008
Decline to NW in OW status with MPA	603	7.48	0.79 (0.73–0.87)	< 0.0001	0.75 (0.68–0.81)	< 0.0001	0.73 (0.67–0.80)	< 0.0001
Persistent obesity with MPA	1499	9.02	0.96 (0.90–1.02)	0.2118	1.06 (0.99–1.13)	0.0647	1.00 (0.94–1.06)	0.9347

*APA* active physical activity, *CI* confidence interval, *CRP* c-reaction protein, *CVD* cardiovascular disease, *HR* hazard ratio, *MI* myocardial infarction, *MPA* moderate physical activity, *NW* normal-weight, *OW* overweight

^a^Multivariate cox regression analysis was used to evaluate the associations of CVDs and all-cause mortality risk with trajectory groups, adjusting for potential confounding factors

^b^Number represented the number of events

^c^Per 1,000 person-years

^d^CVDs included MI and stroke (cerebral infarction, cerebral hemorrhages, and subarachnoid hemorrhage)

^e^Model 1 was a crude model without adjusted covariates

^f^Model 2 was adjusted for age, sex, type of work, seat time, and walking instead of the elevators

^g^Model 3 was further adjusted for educational level, smoking status, drinking status, family per-member monthly income, salt intake, drinking tea status, CRP, and history of diseases (hypertension, diabetes, and hyperlipidemia)

The unadjusted and adjusted cumulative incidence of MI and stroke according to the trajectory groups (Fig. 3). After adjusting covariates, persistent OW with MPA group, decline to OW in obesity status with MPA group, and persistent obesity with MPA group were associated with an increased risk of CVDs compared with persistent NW with MPA group. Different with above results, rising to OW in NW status with MPA group was associated with a decrease risk of MI and persistent obesity with MPA group was not associated with the risk of MI (Fig. 4; Table 1).

### Sensitivity analyses and stratification analyses

Table 2 shows the sensitivity analyses of the association of the trajectory groups with CVDs and all-cause mortality. In the time-depending or competing models, the similar results were obtained.

**Table 2:** The sensitivity analyses of the associations of the trajectory groups with CVDs and all-cause mortality^a^

	Sensitivity 1^b^	*P*	Sensitivity 2^c^	*P*	Sensitivity 3^d^	*P*
	SHR (95% CI)		HR (95% CI)		SHR (95% CI)	
CVDs^e^						
Persistent NW with MPA (reference)	–	–	–	–		
Rising to OW in NW status with MPA	0.97 (0.85–1.11)	0.6513	0.91 (0.80–1.04)	0.1637	0.94 (0.83–1.08)	0.3795
Persistent OW with MPA	1.33 (1.24–1.44)	< 0.0001	1.33 (1.23–1.43)	< 0.0001	1.35 (1.26–1.46)	< 0.0001
Decline to NW in OW status with MPA	1.03 (0.91–1.16)	0.6518	0.97 (0.86–1.09)	0.6068	1.03 (0.91–1.16)	0.6729
Decline to OW in obesity status with MPA	1.24 (1.08–1.42)	0.0024	1.16 (1.01–1.33)	0.0313	1.24 (1.08–1.42)	0.0021
Persistent obesity with MPA	1.55 (1.42–1.70)	< 0.0001	1.58 (1.44–1.73)	< 0.0001	1.60 (1.46–1.75)	< 0.0001
MI						
Persistent NW with MPA (reference)	–	–	–	–		
Rising to OW in NW status with MPA	0.70 (0.49–1.00)	0.0524	0.69 (0.48–0.98)	0.0357	0.71 (0.50–1.01)	0.0556
Persistent OW with MPA	1.29 (1.08–1.54)	0.0045	1.29 (1.08–1.53)	0.0046	1.31 (1.10–1.56)	0.0024
Decline to NW in OW status with MPA	0.85 (0.62–1.16)	0.2915	0.81 (0.59–1.10)	0.1676	0.85 (0.63–1.16)	0.3064
Decline to OW in obesity status with MPA	1.13 (0.81–1.58)	0.4580	1.06 (0.76–1.48)	0.7223	1.13 (0.81–1.58)	0.4619
Persistent obesity with MPA	1.54 (1.25–1.91)	< 0.0001	1.59 (1.29–1.96)	< 0.0001	1.61 (1.30–1.98)	< 0.0001
Stroke						
Persistent NW with MPA (reference)	–	–	–	–		
Rising to OW in NW status with MPA	1.02 (0.89–1.18)	0.7796	0.95 (0.83–1.10)	0.4892	0.99 (0.86–1.14)	0.8361
Persistent OW with MPA	1.34 (1.23–1.45)	< 0.0001	1.33 (1.23–1.44)	< 0.0001	1.36 (1.25–1.47)	< 0.0001
Decline to NW in OW status with MPA	1.07 (0.94–1.22)	0.3185	1.01 (0.88–1.15)	0.9416	1.07 (0.94–1.21)	0.3402
Decline to OW in obesity status with MPA	1.26 (1.09–1.47)	0.0023	1.19 (1.02–1.38)	0.0234	1.27 (1.09–1.47)	0.0017
Persistent obesity with MPA	1.55 (1.40–1.72)	< 0.0001	1.57 (1.43–1.73)	< 0.0001	1.59 (1.44–1.76)	< 0.0001
All-cause mortality						
Persistent NW with MPA (reference)	–	–	–	–		
Rising to OW with MPA in NW status with APA	–	–	0.74 (0.67–0.81)	< 0.0001	–	–
Persistent OW with MPA	–	–	0.93 (0.88–0.98)	0.0036	–	–
Decline to NW in OW status with MPA	–	–	0.75 (0.69–0.82)	< 0.0001	–	–
Persistent obesity with MPA	–	–	1.02 (0.95–1.08)	0.6192	–	–

^a^Multivariate cox regression analysis was used to evaluate the association of CVDs and all-cause mortality risk with trajectory groups, adjusting for potential confounding factors. HR calculated by cox regression adjusting age, sex, type of work, seat time, walking instead of the elevators, educational level, smoking status, drinking status, family per-member monthly income, salt intake, drinking tea status, CRP, and history of diseases (hypertension, diabetes, and hyperlipidemia)

^b^Considering non-CVD events death as a competing risk event rather than a censoring event, the association of all-cause mortality risk with trajectory groups was evaluated using competing risk model

^c^The association of all-cause mortality risk with trajectory groups was evaluated using time-depending model. Age, type of work, seat time, walking instead of the elevators, smoking status, drinking status, family per-member monthly income, salt intake, drinking tea status, CRP, and history of diseases (hypertension, diabetes, and hyperlipidemia) were updated during 2006–2016. Sex and educational level were time-invariant variables

^d^The association of all-cause mortality risk with trajectory groups was evaluated using time-depending competing risk model

^e^CVDs included MI and stroke (cerebral infarction, cerebral hemorrhages, and subarachnoid hemorrhage)

*APA* active physical activity, *CI* confidence interval, *CRP* c-reaction protein, *CVD* cardiovascular disease, *HR* hazard ratio, *MI* myocardial infarction, *MPA* moderate physical activity, *NW* normal-weight, *OW* overweight, *SHR* sub-distribution hazard ratio

In the stratified analyses, compared with persistent NW with MPA group, the associations of rising to OW in NW status with MPA group and persistent OW with MPA group with all-cause mortality were stronger in age ≥ 65 years than in age < 65 years; however, decline to NW in OW status with MPA group and persistent obesity with MPA group with all-cause mortality were stronger in age < 65 years than in age ≥ 65 years (Additional file 1: Table S3). There were significant interactions of age (< 65 years old, ≥ 65 years old) in relationship trajectory groups with all-cause mortality, but no interactions were found for gender in relationship trajectory groups with CVDs, MI, stroke, and all-cause mortality (*P* interaction > 0.05 for all) (Additional file 1: Table S3).

## Discussion

In our prospective cohort study of Chinese adults followed biennially from 2006 to 2019, we examined the development of different trajectories of joint phenotypes of BMI and PA during 2006 to 2016, and assessed the associations between these trajectories and risks of CVDs (including MI and stroke) and all-cause mortality. Within the present study, we identified six trajectories and five trajectories using the group-based trajectory analysis for predicting incident CVDs and all-cause mortality, respectively. Compared with persistent NW with MPA group, participants were at 30.8% and 54.5% higher risk of CVDs in the persistent OW with MPA and persistent obesity with MPA trajectory group respectively, while at no increased risk of all-cause mortality in all trajectory groups. These findings were overall robust in sensitivity analyses.

Weight trajectory has been focused on multiple studies, is related to the risk of coronary heart disease [30, 31], type 2 diabetes [32, 33], blood glucose metabolism [34] and cancers [35]. The present study was the first to assess the links between combined BMI and PA phenotype trajectories with the risk of CVDs and all-cause mortality in Chinese adults. Identifying groups of individuals following similar patterns in distinct trajectories might be useful because it may help to identify different pathways by which OW and obesity development and the mechanisms underlying increasing trends. In addition, this allowed us insight in developing early interventions target specific subgroups based on these distinct trajectories [36].

There was a large cohort study described that waist circumference trajectories (including moderate stable, moderate-high stable and high stable group) were associated with risk of CVDs including stroke and MI [37]. A previously systematic review presented that excess BMI with PA was associated with an increased risk of CVDs, which is consistent with our findings [38]. From the present study, the persistent OW with MPA and persistent obesity with MPA trajectories group were associated with an increased risk of CVDs, suggested that persisting OW or obesity could result in CVDs regardless of PA levels. Although PA can have a positive effect on the cardiovascular system [39], moderate exercise intervention may be ineffective in patients with persistent obesity or overweight. A possible reason presented that severe impairment vascular function attributing from persisting obesity or overweight might not be reversed. Similarly, persisting at high levels of long-term BMI trajectories was strongly associated with cardio-metabolic traits [40]. Previous study demonstrated that participants with obesity but metabolic healthy (MH) were associated with an increased risk for MI [24] compared with MH-NW, which suggested an important influence of obesity or overweight on CVDs. A possible mechanism also demonstrated that it is difficult to lose fat when we get fatter, because our body makes more strongly energy compensation that burned during PA [41]. Therefore, it is importance to maintain healthy weight for decreasing CVDs risk.

Moreover, the decreased risk of all-cause mortality were observed in rising to OW with MPA in NW status with APA, persistent OW with MPA and decline to NW in OW status with MPA trajectories group, this may suggest the existence of the “obesity paradox” [42]. It is possibility participants with MPA might be a state of MH in the present study, because PA was associated with levels of cardiorespiratory and metabolic fitness [43]. Previous studies also presented that participants with MH and obesity (MHO) or MH and overweight (MHOW) was not associated with all-cause mortality [44–46], which suggested MH status might be associated with decrease all-cause mortality. Metabolic phenotypes were associated with body fat distribution patterns including visceral and ectopic fat, which might indirectly result in development of diseases [47]. A possible fat distribution of participants with MHO or MHOW is a high percentage of gluteofemoral and leg fat mass other than accumulation of visceral fat [48]. Additionally, we previously found that MHOW was the healthiest metabolic phenotype [23], which also suggested the importance of association among metabolic factors, lifestyle factors, and diseases.

Our study is strengthened by the use of large prospective cohort study, including approximately 100,000 participants who were followed-up for more than 13 years, allowed us to perform the joint analyses with sufficient statistical power. The prospective design and high follow-up rates minimized the potential for recall bias and loss to follow-up. Besides, we firstly evaluated trajectories of joint phenotypes using the GBTM and conducted a series of sensitivity analyses to show the robustness of the findings. The GBTM allows us to identify multi-unobserved subpopulation, and examine differences in change among unobserved subpopulations [27].

There were several limitations about this present study. Firstly, information on PA level was mainly self-reported, thus measurement bias could not be completely avoidable. However, the self-reported PA questionnaire was validated and demonstrated fairly reliability [26, 49]. Secondly, we excluded participants with less than 3 times measurements of joint phenotypes during 2006 to 2016, due to loss to follow-up including occurring outcomes or other reasons, which might be loss information of partial participants, underestimating the effects of trajectories on outcomes. However, we included 98% of samples in final analysis, and the impact of excluding above participants on the observed association between trajectories and outcomes could be small. Thirdly, we only used BMI to define participants as overweight or obesity. While BMI is widely used in clinical practice, it may be an imperfect measure. And due to the lack of longitudinal measures on indices of fat distribution, we are unable to account for peripheral obesity and abdominal obesity. Fourthly, this study was not a national representative sample, and the racially-homogenous cohort of East Asian ancestry could limit generalizability, the result need to be interpreted cautiously. Fifthly, due to the largest number of participants with MPA, we identified only six and five trajectories of MPA. We cannot estimate the long-term trajectory of obese or overweight patients with high levels of PA and its relationship with CVDs. Finally, the group-based trajectory analysis identifies the unknown population distribution of trajectories, so the trajectory groups should be considered as clusters of individuals following approximately the same trajectory for homogeneity may be theoretically unrealistic within-class [28].

## Conclusions

In the large prospective cohort, six long-term trajectories for CVDs and five trajectories for all-cause mortality of joint phenotypes of BMI and PA were identified and were associated with CVDs and all-cause mortality. Participants were at a higher risk of CVDs in the “persistent OW with MPA” and “persistent obesity with MPA” trajectories group. Our results suggested that persisting OW and obesity participants with MPA may not decrease the risk of CVDs in the trajectory group. We additionally observed the decreased risk of all-cause mortality in trajectories group, suggesting the potential existence of “obesity paradox”.

## Supplementary Information

**Additional file 1: Table S1**. Baseline characteristics of participants according trajectory groups for CVDs outcome. **Table S2**. Baseline characteristics of participants according trajectory groups for all-cause mortality outcome. **Table S3**. The association of the trajectory groups with CVDs and all-cause mortality stratified subgroups.

## Data Availability

The data underlying this article are available from the corresponding author on reasonable request.

## Abbreviations

AvePP: Average posterior probability
AIC: Akakike information criteria
APA: Active physical activity
BIC: Bayesian information criterion
BMI: Body mass index
CI: Confidence interval
CRP: C-reactive protein
CVD: Cardiovascular disease
FBG: Fasting blood glucose
GBTM: Group-based trajectory model
HC: Hip circumference
HDL-C: High-density lipoprotein cholesterol
HR: Hazard ratio
IQR: Interquartile range
LDL-C: Low-density lipoprotein cholesterol
MI: Myocardial infarction
MPA: Moderate active physical activity
NW: Normal weight
NWAPA: Normal-weight and active physical activity
NWMPA: Normal-weight and moderate physical activity
NWIPA: Normal-weight and inactive physical activity
OAPA: Obesity and active physical activity
OIPA: Obesity and inactive physical activity
OMPA: Obesity and moderate physical activity
OW: Overweight
OWAPA: Overweight and active physical activity
OWIPA: Overweight and inactive physical activity
OWMPA: Overweight and moderate physical activity
PA: Physical activity
TC: Total cholesterol
TG: Triglycerides
WC: Waist circumference
