# Supplementary material for: Moderate physical activity may not decrease the risk of cardiovascular disease in persistently overweight and obesity adults
Source: J Transl Med. 2022 Jan 28;20:45. doi: 10.1186/s12967-021-03212-7 (PMC8796584; doi:10.1186/s12967-021-03212-7)
Supplement: Supplementary file 1 — Additional file 1: Table S1. Baseline characteristics of participants according trajectory groups for CVDs outcome. Table S2. Baseline characteristics of participants according trajectory groups for all-cause mortality outcome. Table S3. The association of the trajectory groups with CVDs and all-cause mortality stratified subgroups. [file 12967_2021_3212_MOESM1_ESM.docx]

Table S1. Baseline characteristics of participants according trajectory groups for CVDs outcome^a^

| **Variable** | **Rising to OW in NW status with MPA (n=7,306)** | **Persistent NW with MPA (n=27,629)** | **Persistent OW with MPA (n=30,087)** | **Decline to NW in OW status with MPA (n=6,936)** | **Decline to OW in obesity status with MPA (n=4,315)** | **Persistent obesity with MPA (n=11,868)** | ***P*** |
| --- | --- | --- | --- | --- | --- | --- | --- |
| Age, year, median (IQR) | 48.65 (14.78) | 50.68 (15.13) | 51.35 (14.09) | 52.59 (13.74) | 52.44 (15.25) | 51.41 (15.26) | <0.0001 |
| Men, no. (%) | 5454 (74.65) | 21112 (76.41) | 24884 (82.71) | 5514 (79.50) | 3496 (81.02) | 9340 (78.70) | <0.0001 |
| Physical labour, no. (%) | 6819 (93.46) | 25318 (91.75) | 27806 (92.59) | 6371 (92.04) | 3969 (92.17) | 11008 (92.92) | <0.0001 |
| Senior high school or above, no. (%) | 1516 (20.75) | 6047 (21.89) | 5816 (19.33) | 1318 (19.00) | 855 (19.82) | 2331 (19.64) | <0.0001 |
| Seat time, ≥ 4 hours, no. (%) | 1652 (22.67) | 6884 (24.95) | 7321 (24.39) | 1678 (24.24) | 1153 (26.75) | 3063 (25.86) | <0.0001 |
| Walking, no. (%) | 6683 (91.57) | 25522 (92.50) | 27856 (92.80) | 6445 (93.08) | 4006 (92.97) | 10919 (92.19) | 0.0018 |
| Family per-member income > 1000 Yuan/month, no. (%) | 445 (6.09) | 1885 (6.82) | 1993 (6.62) | 494 (7.12) | 282 (6.54) | 784 (6.61) | 0.1831 |
| Current smoking, no. (%) | 2515 (34.47) | 9755 (35.35) | 10466 (34.84) | 2197 (31.69) | 1361 (31.56) | 3840 (32.40) | <0.0001 |
| Current drinking, no. (%) | 2728 (37.35) | 10220 (37.02) | 11741 (39.07) | 2475 (35.70) | 1623 (37.64) | 4322 (36.43) | <0.0001 |
| Salt intake > 12g/d, no. (%) | 710 (9.72) | 2621 (9.49) | 3292 (10.95) | 691 (9.97) | 506 (11.74) | 1531 (12.91) | <0.0001 |
| History of hypertension, no. (%) | 443 (6.07) | 1803 (6.53) | 3859 (12.84) | 917 (13.23) | 824 (19.11) | 2348 (19.80) | <0.0001 |
| History of diabetes, no. (%) | 129 (1.77) | 539 (1.95) | 902 (3) | 216 (3.11) | 184 (4.27) | 460 (3.88) | <0.0001 |
| History of hyperlipidemia, no. (%) | 197 (2.70) | 846 (3.06) | 1787 (5.94) | 452 (6.52) | 384 (8.90) | 1121 (9.45) | <0.0001 |
| Drinking tea, no. (%) | 1688 (23.12) | 6591 (23.88) | 7765 (25.84) | 1645 (23.75) | 1220 (28.31) | 3117 (26.29) | <0.0001 |
| SBP, mmHg, median (IQR) | 120 (23.70) | 120.70 (28) | 130 (22) | 130 (23.30) | 135 (29.30) | 133 (30) | <0.0001 |
| DBP, mmHg, median (IQR) | 80 (15.30) | 80 (16) | 81.30 (10.70) | 80.70 (10.70) | 87.30 (15) | 86.70 (16) | <0.0001 |
| WC, cm, median (IQR) | 83 (10) | 80 (10) | 89 (9.50) | 87 (9.30) | 95 (10) | 96 (11) | <0.0001 |
| HC, cm, median (IQR) | 94 (8) | 92 (7.60) | 99 (8) | 97 (8) | 104 (9) | 105 (10) | <0.0001 |
| TG, mmol/L, median (IQR) | 1.12 (0.84) | 1.05 (0.73) | 1.40 (1.11) | 1.31 (1.01) | 1.63 (1.32) | 1.64 (1.30) | <0.0001 |
| TC, mmol/L, median (IQR) | 4.85 (1.28) | 4.84 (1.27) | 4.97 (1.32) | 5 (1.31) | 5.03 (1.34) | 5.02 (1.32) | <0.0001 |
| LDL-C, mmol/L, median (IQR) | 2.30 (1.01) | 2.29 (1) | 2.40 (0.98) | 2.40 (0.94) | 2.40 (0.99) | 2.39 (1.01) | <0.0001 |
| HDL-C, mmol/L, median (IQR) | 1.54 (0.49) | 1.56 (0.49) | 1.48 (0.46) | 1.52 (0.48) | 1.46 (0.46) | 1.43 (0.46) | <0.0001 |
| FBG, mmol/L, median (IQR) | 5.03 (0.93) | 5.01 (0.93) | 5.18 (1.09) | 5.20 (1.15) | 5.32 (1.20) | 5.23 (1.13) | <0.0001 |
| CRP, mg/L, median (IQR) | 0.60 (1.50) | 0.55 (1.32) | 0.81 (1.69) | 0.80 (1.65) | 1.10 (2.09) | 1.21 (2.28) | <0.0001 |

^a^Data represent median (interquartile range [IQR] 25%-75%) or number and percentage.

CRP, C-reactive protein; CVDs, cardiovascular diseases; DBP, diastolic blood pressure; FBG, fasting blood glucose; HC, hip circumference; HDL-C, high-density lipoprotein cholesterol; LDL-C, low-density lipoprotein cholesterol; MPA, moderate physical activity; NW, normal weight; OW, overweight; SBP, systolic blood pressure; TC, total cholesterol; TG, triglycerides; WC, waist circumference.

Table S2. Baseline characteristics of participants according trajectory groups for all-cause mortality outcome^a^

| **Variable** | **Rising to OW with MPA in NW status with APA (n=28,405)** | **Persistent NW with MPA (n=7,853)** | **Persistent OW with MPA (n=32,304)** | **Decline to NW in OW status with MPA (n=6,855)** | **Persistent obesity with MPA (n=14,319)** | ***P*** |
| --- | --- | --- | --- | --- | --- | --- |
| Age, year, median (IQR) | 50.78 (15.25) | 48.87 (14.70) | 51.52 (14.32) | 52.82 (13.64) | 51.83 (15.32) | <0.0001 |
| Men, no. (%) | 21765 (76.62) | 5903 (75.17) | 26773 (82.88) | 5459 (79.64) | 11324 (79.08) | <0.0001 |
| Physical labour, no. (%) | 26042 (91.79) | 7325 (93.40) | 29863 (92.63) | 6294 (92.00) | 13267 (92.82) | <0.0001 |
| Senior high school or above, no. (%) | 6178 (21.75) | 1608 (20.48) | 6220 (19.26) | 1295 (18.89) | 2776 (19.39) | <0.0001 |
| Seat time, ≥ 4 hours, no. (%) | 7067 (24.91) | 1763 (22.50) | 7928 (24.59) | 1668 (24.39) | 3707 (25.94) | <0.0001 |
| Walking, no. (%) | 26243 (92.52) | 7187 (91.65) | 29938 (92.87) | 6375 (93.16) | 13184 (92.27) | 0.0007 |
| Family per-member income > 1000Yuan/month, no. (%) | 1941 (6.83) | 467 (5.95) | 2149 (6.65) | 503 (7.34) | 919 (6.42) | 0.0067 |
| Current smoking, no. (%) | 10045 (35.40) | 2711 (34.58) | 11181 (34.66) | 2166 (31.61) | 4636 (32.42) | <0.0001 |
| Current drinking, no. (%) | 10504 (37.01) | 2925 (37.27) | 12563 (38.93) | 2438 (35.59) | 5220 (36.47) | <0.0001 |
| Salt intake > 12g/d, no. (%) | 2704 (9.53) | 764 (9.73) | 3550 (11) | 692 (10.10) | 1832 (12.80) | <0.0001 |
| History of hypertension, no. (%) | 1917 (6.76) | 502 (6.40) | 4374 (13.56) | 939 (13.70) | 2892 (20.21) | <0.0001 |
| History of diabetes, no. (%) | 579 (2.04) | 141 (1.80) | 1030 (3.19) | 241 (3.52) | 566 (3.95) | <0.0001 |
| History of hyperlipidemia, no. (%) | 887 (3.12) | 225 (2.87) | 1999 (6.19) | 458 (6.68) | 1368 (9.56) | <0.0001 |
| Drinking tea, no. (%) | 6764 (23.84) | 1824 (23.24) | 8379 (25.79) | 1643 (24.00) | 3779 (26.43) | <0.0001 |
| SBP, mmHg, median (IQR) | 120.70 (28) | 120.70 (25.30) | 130 (24) | 130 (24.70) | 134.70 (29.30) | <0.0001 |
| DBP, mmHg, median (IQR) | 80 (15.70) | 80 (16.70) | 81.30 (10) | 80.70 (10.70) | 87.30 (16) | <0.0001 |
| WC, cm, median (IQR) | 80 (10) | 83 (10) | 89 (9) | 87 (10) | 91 (11) | <0.0001 |
| HC, cm, median (IQR) | 92 (7.50) | 94 (8) | 99 (8) | 97 (8) | 105 (10) | <0.0001 |
| TG, mmol/L, median (IQR) | 1.05 (0.74) | 1.14 (0.86) | 1.41 (1.12) | 1.31 (1.02) | 1.65 (1.31) | <0.0001 |
| TC, mmol/L, median (IQR) | 4.84 (1.27) | 4.85 (1.28) | 4.99 (1.32) | 5 (1.31) | 5.02 (1.32) | <0.0001 |
| LDL-C, mmol/L, median (IQR) | 2.29 (1.00) | 2.30 (1.03) | 2.40 (0.99) | 2.40 (0.94) | 2.40 (1.01) | <0.0001 |
| HDL-C, mmol/L, median (IQR) | 1.56 (0.49) | 1.53 (0.49) | 1.48 (0.47) | 1.52 (0.48) | 1.43 (0.46) | <0.0001 |
| FBG, mmol/L, median (IQR) | 5.01 (0.94) | 5.04 (0.93) | 5.20 (1.08) | 5.20 (1.19) | 5.25 (1.12) | <0.0001 |
| CRP, mg/L, median (IQR) | 0.56 (1.33) | 0.60 (1.54) | 0.85 (1.76) | 0.80 (1.70) | 1.21 (2.28) | <0.0001 |

^a^Data represent median (interquartile range [IQR] 25%-75%) or number and percentage.

APA, active physical activity; CRP, C-reactive protein; CVDs, cardiovascular diseases; DBP, diastolic blood pressure; FBG, fasting blood glucose; HC, hip circumference; HDL-C, high-density lipoprotein cholesterol; LDL-C, low-density lipoprotein cholesterol; MPA, moderate physical activity; NW, normal weight; OW, overweight; SBP, systolic blood pressure; TC, total cholesterol; TG, triglycerides; WC, waist circumference.

Table S3. The association of the trajectory groups with CVDs and all-cause mortality stratified subgroups^a^

|  | **Age < 65 years** | **Age ≥ 65 years** | **Female** | **Male** |
| --- | --- | --- | --- | --- |
| **CVDs^b^** |  |  |  |  |
| Persistent NW with MPA **(reference)** | **-** | **-** | **-** | - |
| Rising to OW in NW status with MPA | 0.90 (0.78-1.04) | 0.75 (0.53-1.05) | 0.82 (0.54-1.23) | 0.94 (0.82-1.08) |
| Persistent OW with MPA | 1.29 (1.18-1.40) | 1.29 (1.10-1.50) | 1.38 (1.09-1.74) | 1.29 (1.20-1.40) |
| Decline to NW in OW status with MPA | 0.98 (0.85-1.12) | 0.95 (0.75-1.22) | 0.90 (0.61-1.04) | 0.96 (0.85-1.10) |
| Decline to OW in obesity status with MPA | 1.15 (0.99 -1.35) | 1.04 (0.77 -1.42) | 0.96 (0.63-1.48) | 1.16 (1.00 -1.34) |
| Persistent obesity with MPA | 1.50 (1.36-1.67) | 1.38 (1.12-1.70) | 1.64 (1.26-2.14) | 1.52 (1.38-1.67) |
| ^c^*P* for Heterogeneity | 0.5842 |  | 0.4936 |  |
| **MI** |  |  |  |  |
| Persistent NW with MPA **(reference)** | - | - | - | - |
| Rising to OW in NW status with MPA | 0.71 (0.49-1.04) | 0.30 (0.09-0.96) | 0.46 (0.11-2.01) | 0.69 (0.48-1.00) |
| Persistent OW with MPA | 1.25 (1.02-1.52) | 1.22 (0.86-1.73) | 1.54 (0.83-2.88) | 1.24 (1.03-1.48) |
| Decline to NW in OW status with MPA | 0.84 (0.59-1.20) | 0.68 (0.37-1.27) | 0.69 (0.23-2.07) | 0.80 (0.58-1.10) |
| Decline to OW in obesity status with MPA | 1.00 (0.68-1.47) | 1.08 (0.55-2.12) | 0.76 (0.22-2.65) | 1.07 (0.76-1.52) |
| Persistent obesity with MPA | 1.45 (1.14-1.84) | 1.52 (0.98-2.37) | 1.77 (0.88-3.55) | 1.49 (1.19-1.86) |
| *P* for Heterogeneity | 0.8062 |  | 0.7857 |  |
| **Stroke** |  |  |  |  |
| Persistent NW with MPA **(reference)** | - | - | - | - |
| Rising to OW in NW status with MPA | 0.93 (0.80-1.09) | 0.86 (0.60-1.22) | 0.87 (0.57-1.34) | 0.99 (0.85-1.15) |
| Persistent OW with MPA | 1.29 (1.18-1.41) | 1.31 (1.10-1.56) | 1.36 (1.05-1.75) | 1.30 (1.19-1.42) |
| Decline to NW in OW status with MPA | 1.00 (0.86-1.17) | 1.04 (0.80-1.31) | 0.93 (0.62-1.40) | 1.00 (0.87-1.15) |
| Decline to OW in obesity status with MPA | 1.18 (0.99-1.39) | 1.07 (0.77-1.49) | 1.00 (0.63-1.58) | 1.18 (1.01-1.39) |
| Persistent obesity with MPA | 1.07 (0.77-1.49) | 1.33 (1.06-1.68) | 1.64 (1.23-2.18) | 1.52 (1.36-1.69) |
| *P* for Heterogeneity | 0.5827 |  | 0.6694 |  |
| **All-cause mortality** |  |  |  |  |
| Persistent NW with MPA **(reference)** | - | - | - | - |
| Rising to OW with MPA in NW status with APA | 0.65 (0.58-0.74) | 0.67 (0.58-0.78) | 0.77 (0.56-1.05) | 0.71 (0.64-0.79) |
| Persistent OW with MPA | 0.87 (0.81-0.93) | 0.92 (0.85-0.99) | 0.96 (0.80-1.15) | 0.91 (0.86-0.96) |
| Decline to NW in OW status with MPA | 0.84 (0.74-0.95) | 0.59 (0.52-0.67) | 0.66 (0.49-0.89) | 0.74 (0.67-0.81) |
| Persistent obesity with MPA | 0.92 (0.84-1.01) | 1.00 (0.91 -1.09) | 1.13 (0.92 -1.38) | 0.98 (0.92-1.05) |
| *P* for Heterogeneity | 0.0001 |  | 0.6116 |  |

Data represent HR (95%CI).

^a^Model was adjusted age, sex, type of work, seat time, walking instead of the elevators, educational level, smoking status, drinking status, family per-member monthly income, salt intake, drinking tea status, CRP, and history of diseases (hypertension, diabetes, and hyperlipidemia).

^b^CVDs included MI and stroke.

*^c^P* for heterogeneity was attained from the likelihood ratio test.

APA, active physical activity; CI, confidence interval; CRP, C-reactive protein; CVDs, cardiovascular diseases; HR, hazard ratio; MI, myocardial infarction; MPA, moderate physical activity; NW, normal weight; OW, overweight.
